# Could the Sasang constitution itself be a risk factor of abdominal obesity?

**DOI:** 10.1186/1472-6882-13-72

**Published:** 2013-04-02

**Authors:** Eunsu Jang, Younghwa Baek, Kihyun Park, Siwoo Lee

**Affiliations:** 1 Division of Constitutional Medicine/Diagnosis Research Group, Korea Institute of Oriental Medicine, 1672 Yuseongdae-ro, Yuseong-gu, 305-811, Daejeon, Republic of Korea

**Keywords:** Abdominal obesity, Sasang constitutional medicine, Prevalence, Waist circumference, Risk factor

## Abstract

**Background:**

Abdominal obesity (AO) is a medical condition in which excess body fat accumulates in the abdomen. It may cause adverse effects on health and result in reduced life expectancy or increased health problems. While various genetic approaches have explained the risks of AO in Western society, the Sasang constitution (SC) has been identified as a risk factor in Korean medicine. Different SC types are associated with different fat distribution, body shapes and susceptibility to diseases. We evaluated whether the SC type could be a risk for AO in a cross-sectional study among Koreans.

**Methods:**

In total, 2,528 subjects aged over 30 years were recruited from 23 medical clinics. We collected waist circumference (WC), weight, height, and some clinical information for AO from the subjects. A Chi-square test and a one-way ANOVA were performed according to SC type (p < .05), while multiple logistic regression was used to produce odds ratios (ORs).

**Results:**

The rates of AO in Tae-eumin (TE), Soeumin (SE), and Soyangin (SY) types were 63.7%, 14.7%, and 32.8% in males and 84.8%, 41.7%, and 52.8% in females, respectively. The TE type was associated with increased AO prevalence compared with the SE and SY types in males (OR 1.79; 95% CI 1.02–3.15, p = 0.044 and OR 1.74; 95% CI 1.18–2.58, p = 0.006, respectively) and females (OR 1.51; 95% CI 1.03–2.23, p = 0.037 and OR 1.88; 95% CI 1.32–2.68, p < 0.001, respectively) after adjusting for age, BMI, hypertension, diabetes mellitus, hypertriglyceridemia, and low HDL cholesterol.

**Conclusions:**

This study suggested that SC, particularly the TE type, might be significantly and independently associated with AO and could be considered a risk factor in predicting AO.

## Background

Obesity is a medical condition in which excess body fat has accumulated to the extent that it may have an adverse effect on health, leading to reduced life expectancy and/or increased health problems [1]. Obesity, especially abdominal obesity (AO), is a substantial risk factor for hypertension, metabolic disease, cardiovascular disease, gallbladder disease, osteoarthritis, and sleep apnea [2].

Furthermore, the waist circumference (WC) plays pivotal roles in those diseases because of its central fat distribution [3-7]. Therefore, the World Health Organization (WHO) has classified obese guide according to not only body mass index (BMI) but also WC [8] and the National Cholesterol Education Program Adult Treatment Panel III also has defined AO as one of important factors for metabolic syndrome [9]. Eventually, the WHO has identified that human beings should overcome AO in the 21st century and observed that the wait for a cure may take a long time [10]. The fundamental cause of AO is presumed to be a combination of environmental factors (inappropriate eating and physical inactivity) and the organism’s genes, such as the 5-HT2A gene and glucocorticoid receptor (GR) gene in Western society [11-15].

Sasang constitutional medicine (SCM) is a tailored Korean traditional medicine that classifies human beings into four constitutions: Taeyangin (TY), Soyangin (SY), Tae-eumin, (TE), and Soeumin (SE) [16]. Each constitution is classified by characteristics of body shape, face, voice, and psychological and physiological aspects [17,18], and those characteristics are different from one constitution to another [19]. Therefore, each constitution has a different susceptibility to pathology and several chronic diseases. Hypertension and diabetes mellitus (DM) were revealed to be associated with a specific Sasang constitution (SC) [19-23].

According to SCM theory, lung hypo-function and liver hyper-function are related to a large WC, and the TE type is associated with hyperactive liver function and a developed waist area [16,17]. We hypothesize that SC could be a risk for AO. Several family studies have been conducted to investigate the genetic evidence for SC, and they found that SC could be not only inherent but also a risk factor for obesity [24,25]. However, there has been no clinical study to show that SC could a risk factor for AO.

In this study, we present indirect evidence of whether SC could be a risk factor for AO among Koreans.

## Methods

This was a cross-sectional study conducted from Nov. 2007 to Jul. 2011 in 23 Korean medical clinics (KMCs).

### Study subjects and size

**Figure 1:**
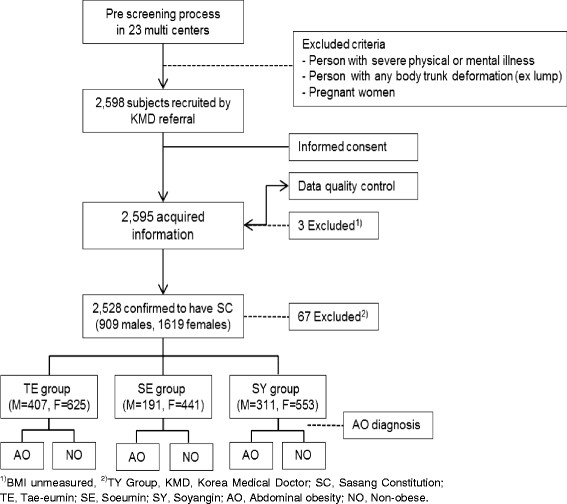
The flow chart of the study.

The study size was assumed from a Bernoulli distribution. We calculated a sample size of at least 600 subjects of each constitution on the basis of a 95% confidence interval and a 4% margin of error. The eligible subjects were recruited from KMCs among individuals over 30 years old whose constitution had been confirmed by experts in SC. Individuals who could not understand and follow the researcher’s indication or keep their measurement posture because of severe physical/mental illness were excluded. The detailed researcher’s indication and the subjects’ measurement posture were described in Jang’s study [26]. The subjects with body deformation such as lump or congenital malformation in the measurement location, or pregnant women were also excluded. A total of 2,598 subjects (931 males and 1,667 females) were recruited from 23 KMCs. Three of them were excluded because of missing data. A total of 67 TY types were also excluded because of their low proportion in the Korean population. In total, 2,528 subjects (909 males and 1,619 females) were included in the final analysis. A flowchart of the study design is shown in Figure 1.

This study was approved by the Korea Institute of Oriental Medicine (KIOM) Institutional Review Board (I-0910/02-001). Written informed consent for participation in this study was obtained from each of the subjects.

### Sasang constitutional diagnosis

An SCM expert at each hospital diagnosed individual SC types. For accurate diagnoses, we strictly adhered to defined qualifications of the experts and subject criteria. The SCM experts had more than 5 years of experience in clinical practice. The administration of constitution-specific pharmaceuticals was used as an additional method to confirm the subject SC. A more detailed procedure of diagnosing SC was described in Song’s study [27].

### Data collection

We collected WC measurements to determine the AO prevalence according to SC. We also collected data on age, sex, body mass index (BMI), and blood pressure and blood samples to control for the influence of confounding factors, which are important risk factors for AO [28,29]. WC was measured around the level of the umbilical scar of the subjects, who took their upper clothing off and stood in an erect posture with their arms folded in front of their chest [26]. BMI was indirectly calculated through the weight and height, and the blood pressure was measured from each subject’s left upper arm after enough rest. To reduce measurement bias among the KMCs, all instructors were educated by KIOM at least once per year, and KIOM monitored the progress of the data collection. All instructors followed a standard operation procedure (SOP) that was developed for the “Korea Constitution Multicenter Study” [26,30].

Blood samples were collected after more than 12 hours of fasting, and the fasting blood glucose, triglyceride (TG) and HDL cholesterol levels were tested by an authorized institution.

### Diagnostic criteria

Hypertension was diagnosed by following the guidelines of the 7th Report of the JNC as ≥ 90 mmHg for diastolic pressure, ≥140 mmHg for systolic pressure or taking medicine for the treatment of high blood pressure [31]. DM was diagnosed with the ADA criteria as ≥126 mg/dl of fasting plasma glucose or taking medicine for the treatment of DM [32]. Hypertriglyceridemia was diagnosed as TG ≥150 mg/dl, and low HDL cholesterol was diagnosed as HDL cholesterol <40 mg/dl in males and <50 mg/dl in females. To diagnose AO, we followed the WHO Report of Asia-Pacific guideline of WC ≥ 90 cm for males and ≥ 80 cm for females [33].

### Statistical analysis

Considering the influence of sexual differences, all analyses were separately conducted in males and females. A one-way ANOVA was used to compare continuous variables (Scheffé’s post-hoc analysis). A Chi-square test was performed to compare the prevalence of AO according to SC. Multiple logistic regression was used to calculate odds ratios (ORs) for AO. To evaluate whether the SC could be a risk factor for AO, covariant variables, including age, BMI, hypertension, DM, hypertriglyceridemia, and low HDL cholesterol, were considered. We conducted all of the analyses using SPSS 17.0 software (SPSS Inc., Chicago, IL). The statistical levels of significance were considered to be p values <0.05.

## Results

### General characteristics

The distribution of SC into TE, SE, and SY types was 44.8%, 21%, and 34.2% in males and 38.6%, 27.2%, and 34.2% in females, respectively. The subjects’ general characteristics, including age, body mass index, systolic blood pressure, diastolic blood pressure, fasting blood glucose, TG, HDL cholesterol, and WC, are shown in Table 1 according to gender and constitution.

**Table 1:** The Characteristics of subjects according to gender and constitution

**Variables**	**Constitution type**			***P *value**
	**TE**	**SE**	**SY**	
Male				
Number (%)	407 (44.8)	191 (21)	311 (34.2)	
Age (years)	52.5 ± 12.1^b^	48.5 ± 12.2^a,b^	52.8 ± 12.4^a^	<0.001
BMI (kg/m^2^)	25.8 ± 2.8^b,c^	21.9 ± 2.5^a,b^	23.7 ± 2.6^a,c^	<0.001
Systolic blood pressure (mmHg)	127.2 ± 14.5^b,c^	120.4 ± 14.6^b^	122.8 ± 14.5^c^	<0.001
Diastolic blood pressure (mmHg)	81.7 ± 10.5^b^	77.4 ± 11.3^a,b^	79.7 ± 10.1^c^	<0.001
Fasting blood glucose (mg/dl)	105.6 ± 31.2	100.8 ± 33.7	105.8 ± 33.6	0.17
TG (mg/dl)	170.0 ± 106.4^b^	126.0 ± 67.1^a,b^	158.3 ± 106.0^c^	<0.001
HDL cholesterol (mg/dl)	39.1 ± 9.6^b,c^	43.2 ± 9.9^b^	41.8 ± 11.1^c^	<0.001
WC (cm)	92.4 ± 7.4	82.2 ± 7.1	86.5 ± 7.9	<0.001
Female				
Number (%)	625 (38.6)	441 (27.2)	553 (34.2)	
Age (years)	53.6 ± 12.7^b,c^	49.3 ± 12.9^b^	50.19 ± 12.1^c^	<0.001
BMI (kg/m^2^)	25.5 ± 3.0^b,c^	21.5 ± 2.5^a,b^	22.8 ± 2.6^a,c^	<0.001
Systolic blood pressure (mmHg)	122.6 ± 17.2^b,c^	116.2 ± 16.0^b^	117.3 ± 14.6^c^	<0.001
Diastolic blood pressure (mmHg)	78.3 ± 11.7^b,c^	74.2 ± 11.3^b^	75.2 ± 10.5^c^	<0.001
Fasting blood glucose (mg/dl)	101.7 ± 315^b^	92.9 ± 15.6^a,b^	98.0 ± 32.1^c^	<0.001
TG (mg/dl)	137.2 ± 89.4^b,c^	100.8 ± 57.7^a,b^	115.9 ± 68.2^a,c^	<0.001
HDL cholesterol (mg/dl)	45.5 ± 12.3^b,c^	51.0 ± 12.6^a,b^	48.5 ± 12.4^a,c^	<0.001
WC (cm)	88.9 ± 8.8	78.6 ± 8.1	81.2 ± 8.2	<0.001

Data shown are the mean ± SD, unless otherwise indicated. ^a^Soeumin and Soyangin differ significantly, ^b^Soeumin and Tae-eumin differ significantly, ^c^Soyangin and Tae-eumin differ significantly. TE, Tae-eumin; SE, Soeumin; SY, Soyangin; BMI, Body mass index; TG, Triglycerides; WC, Waist circumference.

### The rate of AO according to SC

The rate of AO was 42.8% in males and 62.1% in females. The prevalence of AO differed significantly according to constitution. The male AO rate according to constitution was 63.7% in the TE type, 14.7% in the SE type and 32.8% in the SY type, and the female AO rate according to constitution was 84.8% in the TE type, 41.7% in the SE type and 52.8% in the SY type. The details are shown in Table 2.

**Table 2:** Prevalence of abdominal obesity stratified by gender and constitution

**Variables**	**Constitution type**			**Total**	***P *value**
	**TE**	**SE**	**SY**		
Male					
AO	259 (63.7)	28 (14.7)	102 (32.8)	389 (42.8)	
NO	148 (36.3)	163 (85.3)	209 (67.2)	520 (57.2)	<0.001
Total	407 (100)	191 (100)	311 (100)	909 (100)	
Female					
AO	530 (84.8)	184 (41.7)	292 (52.8)	1006 (62.1)	
NO	95 (15.2)	257 (58.3)	261 (47.2)	613 (37.9)	<0.001
Total	625 (100)	441 (100)	553 (100)	1619 (100)	

Data are shown as the n (%). TE, Tae-eumin; SE, Soeumin; SY, Soyangin; AO, Abdominal obesity; NO, Non-obese.

### ORs for AO before and after adjustment

Table 3 shows a sequentially developed multiple logistic regression model of AO. Model 1 was crude, without adjustment, model 2 was adjusted for age and BMI, and model 3 was adjusted for age, BMI, hypertension, DM, hypertriglyceridemia, and low HDL cholesterol. Because the TE type was assumed to be a more dangerous risk factor than other types, the results were described as SE type versus TE type and SY type versus TE type.

**Table 3:** Adjusted odds ratios and 95% CI for abdominal obesity according to constitution

**Variables**	**Model 1**		**Model 2**		**Model 3**	
	**OR (95% CI)**	***P *value**	**OR (95% CI)**	***P *value**	**OR (95% CI)**	***P *value**
Male						
SE Type:TE Type	1:10.19 (6.5–15.96)	<0.001	1:1.77 (1.01–3.16)	0.045	1:1.79 (1.02–3.15)	0.044
SY Type:TE Type	1:3.59 (2.63–4.9)	<0.001	1:1.72 (1.17–2.53)	0.006	1:1.74 (1.18–2.58)	0.006
Female						
SE Type:TE Type	1:7.79 (5.84–10.4)	<0.001	1:1.65 (1.13–2.41)	0.01	1:1.51 (1.03–2.23)	0.037
SY Type:TE Type	1:4.99 (3.79–6.56)	<0.001	1:1.9 (1.35–2.7)	<0.001	1:1.88 (1.32–2.68)	<0.001

Results from logistic regression analysis, Model 1- crude. Model 2- adjusted for age and BMI. Model 3- adjusted for age, BMI, hypertension, DM, hypertriglyceridemia, and low HDL cholesterol. TE, Tae-eumin; SE, Soeumin; SY, Soyangin; OR, odds ratio; CI, confidence interval.

The result revealed that the TE type was associated with increased AO prevalence compared with SE and SY types in males (model 1, OR 10.19; 95% CI 6.5–15.96, p < 0.001 and OR 3.59; 95% CI 2.63–4.9, p < 0.001, respectively). The TE type remained significantly associated with increased AO prevalence after adjusting for age and BMI (model 2, OR 1.77; 95% CI 1.01-3.16, p = 0.045 and OR 1.72; 95% CI 1.17-2.53, p = 0.006, respectively) and age, BMI, hypertension, DM, hypertriglyceridemia, and low HDL cholesterol (model 3, OR 1.79; 95% CI 1.02-3.15, p = 0.044 and OR 1.74; 95% CI 1.18–2.58, p = 0.006, respectively).

We also found a similar trend in females. The TE type was associated with increased AO prevalence compared with SE and SY types (model 1, OR 7.79; 95% CI 5.84–10.4, p < 0.001 and OR 4.99; 95% CI 3.79–6.56, p < 0.001, respectively), and the TE type remained significantly associated with increased AO prevalence after adjusting for age and BMI (model 2, OR 1.65; 95% CI 1.13–2.41, p = 0.01 and OR 1.9; 95% CI 1.35–2.7, p < 0.001, respectively) and age, BMI, hypertension, DM, hypertriglyceridemia, and low HDL cholesterol (model 3, OR 1.51; 95% CI 1.03-2.23, p = 0.037 and OR 1.88; 95% CI 1.32–2.68, p < 0.001, respectively).

## Discussion

Several studies have suggested that the SC could be associated with some chronic diseases, and it has been recognized as an independent risk factor [19-23]. Recently, a gene-level study also revealed that SC was associated with AO [34]. In this cross-sectional study, we aimed to determine whether the SC could be a risk factor for AO among Koreans.

This study suggested that SC may be a significant and independent risk factor of AO. Specifically, the TE type was associated with increased AO prevalence compared with the SE and SY types in males (OR 1.79; 95% CI 1.02–3.15, and OR 1.74; 95% CI 1.18–2.58, respectively) and females (OR 1.51; 95% CI 1.03–2.23, and OR 1.88; 95% CI 1.32-2.68, respectively), even after adjusting for age, BMI, hypertension, DM, hypertriglyceridemia, and low HDL cholesterol. This result means that the TE type could be more susceptible to AO than other types, which is similar to the trend found for hypertension, DM, and metabolic syndrome, which are associated with SC [19-23].

The results revealed that the WC was different according to SC type, and the TE type had the largest WC, which was similar to other characteristics. The rates of AO were different among SC types, and the rate of AO in the TE type was higher than in other types in both genders. However, because the comparatively larger WC of the TE type may be caused by a relatively higher BMI, age, and several chronic diseases [35], it is necessary to explain the pure influence of SC itself. Accordingly, we calculated ORs for AO with a logistic regression analysis model after adjusting for the influence of potential variables. The multiple logistic regression analysis showed that large differences in the OR between the TE type and other types prior to adjusting for the potential variables (model 1) were still significant in models 2 and 3. Furthermore, this trend was similar in both males and females.

This trend supports the hypothesis that people classified as TE type might have a bigger belly and a higher prevalence rate for AO compared with other types, even if each type has similar physical characteristics. This finding might also be indirect evidence for SCM, which suggests that body shape could develop differently from one constitution to another.

In this study, we tried to enroll a representative Korean population from nationwide centers. We also minimized measurement error by following SOPs [30] and blood testing error by utilizing a qualified institution.

The prevalence rates of AO in this study were relatively high compared with a previous study conducted by the Korean government [36]. For this reason, we assumed that the average age in this study was relatively higher.

Our study has several limitations. Previously, a large Korean family study suggested a significant association of chromosomes 8q11.22-23 and 11q22.1-3 with SC [25], and another study found that SC was associated with AO at the genetic level [34]. Family surveys and gene-level studies could be a good method to demonstrate whether SC is an inherent risk factor for AO. However, because our study had a cross-sectional design, we could not analyze the association between inherent SC and AO. In addition, we did not control for environmental factors, such as meals, lifestyle, and exercise habits, which are acquired risk factors for AO.

We believe that further studies on not only a direct comparison between constitutions considering acquired environmental factors for AO but also on inherent family SC are needed.

## Conclusions

This study suggested that SC, especially the TE type, might be significantly and independently associated with AO. This finding reveals that SC should be considered a risk factor in predicting AO.

## Abbreviations

TE: Tae-eumin; SE: Soeumin; SY: Soyangin; AO: Abdominal obesity; NO: Non-obese; WC: Waist circumference.

## Competing interests

The authors declare that they have no competing interests.

## Authors’ contributions

EJ carried out the qualitative data analysis and drafted the manuscript. YB coordinated the study, participated in data collection, and contributed to the interpretation of data and content of this manuscript. SL conceived of this study, was the Principal Investigator, participated in its design and coordination, and contributed to the interpretation of data and content of this manuscript. KP also participated in the qualitative data analysis. All of the authors critically contributed to the final manuscript and approved the final version.

## Pre-publication history

The pre-publication history for this paper can be accessed here:

http://www.biomedcentral.com/1472-6882/13/72/prepub
